# A porcine circovirus type 2d-based virus-like particle vaccine induces humoral and cellular immune responses and effectively protects pigs against PCV2d challenge

**DOI:** 10.3389/fmicb.2023.1334968

**Published:** 2024-01-11

**Authors:** Kiju Kim, Kyusung Choi, Minna Shin, Tae-Wook Hahn

**Affiliations:** ^1^INNOVAC, Chuncheon, Republic of Korea; ^2^College of Veterinary Medicine and Institute of Veterinary Science, Kangwon National University, Chuncheon, Republic of Korea

**Keywords:** porcine circovirus, virus-like particles, PCV2d-based vaccine, miniature pig, protective immunity

## Abstract

The pathogenic porcine circovirus type 2 (PCV2) leads to significant economic losses in pig production. PCV2d is currently the dominant genotype causing porcine circovirus-associated disease (PCVAD) worldwide. Therefore, development of a recombinant PCV2d-based vaccine is required to elicit complete protection against PCV2d infection. In this study, we generated virus-like particles of PCV2d-based capsid protein (Bac-2dCP) using a baculovirus expression system and evaluated its protective efficacy against PCV2d infection in specific pathogen-free (SPF) pigs. Three-week-old SPF miniature pigs were intramuscularly immunized with purified Bac-2dCP and intranasally challenged with PCV2d at 4 weeks post-vaccination. The Bac-2dCP group showed significantly higher IgG levels and neutralizing antibodies against PCV2b and PCV2d genotypes, as well as increased interferon-γ levels, and increased body weight and average daily weight gain compared with positive (challenged) and negative (unchallenged) controls. In particular, the Bac-2dCP group showed almost complete absence of PCV2d DNA in serum, nasal, and rectal swabs and in lung, lymph node, and kidney tissue samples. However, the positive control group exhibited low levels of neutralizing antibody, and high levels of PCV2 DNA in serum, swab, and tissue samples, resulting in PCV2-associated pathological lesions. The results of this study demonstrated that a recombinant Bac-2dCP vaccine conferred complete protection against a PCV2d challenge in SPF miniature pigs.

## 1 Introduction

Porcine circovirus type 2 (PCV2) is primary agent of porcine circovirus-associated disease (PCVAD), which includes post weaning multisystemic wasting syndrome, porcine dermatitis, nephropathy syndrome, and porcine respiratory disease complex (Opriessnig et al., 2007; Lim et al., 2022). PCV2 infection also renders pigs more susceptible to secondary pathogens such as porcine reproductive and respiratory syndrome virus (PRRSV), porcine parvovirus, and *Mycoplasma* spp. by immunosuppression, resulting in increased mortality. PCVAD incurs an average cost of 3–4 USD (up to 20 USD) per pig in the United States and is therefore recognized as an economically important pathogen in the global swine industry (Gillespie et al., 2009). Because of widespread occurrence of PCV2 on pig farms, vaccination is the only effective method to reduce PCV2 prevalence and thereby control PCVAD (Dvorak et al., 2016).

PCV2 is a small virus approximately 17 nm in diameter, comprising non-enveloped, single-stranded circular DNA of about 1.76 kb in an icosahedral form. PCV2 genotypes are currently classified as PCV2a, PCV2b, PCV2c, PCV2d, and PCV2e by PCV2 open reading frame 2 (ORF2) encoding major capsid protein; PCV2d is currently the most prevalent genotype worldwide (Dupont et al., 2008). The nucleotide sequence similarity of PCV2a ORF2 was 90.8–93.2% with PCV2b and 89.2–92.0% with PCV2d (Zheng et al., 2020). The structural capsid of PCV2 is composed of 60 monomeric capsid proteins that can be self-assembled into PCV2 virus-like particle (VLP) and is known to be an important antigenic determinant that induces neutralizing antibody against PCV2 (Kim et al., 2020). Advantages of PCV2 VLP-based subunit vaccine is very safe, easy preparation, low-cost, high-level of expression and highly effective in PCV2 prevention. Current commercial PCV2 subunit vaccines, Ingelvac CircoFLEX (Boehringer Ingelheim Animal Health), Porcilis PCV (MSD Animal Health) and Circumvent PCV (Merck), are based on PCV2a capsid protein expressed in a baculovirus expression system (Fort et al., 2008; Guo et al., 2022).

After introduction of commercial PCV2a vaccines, a global genotype shift from PCV2a to PCV2b was confirmed in vaccinated herds presenting severe clinical symptoms (Carman et al., 2008). In 2014, a newly emerging PCV2b mutant (PCV2d) was reported in cases of vaccine failure in several countries, including Korea, Brazil, and the United States (Xiao et al., 2012; Salgado et al., 2014; Seo et al., 2014). PCV2d is now the most common genotype causing PCVAD (Opriessnig et al., 2019; Park et al., 2019). This genotype shift is highly likely to occur by persistent PCV2 infection and evasion of the host immune response (Franzo et al., 2016).

It has been proposed that PCV2a-based vaccines cannot provide complete protection against the prevalent PCV2d genotype (Hou et al., 2019; Tseng et al., 2019). In a previous study, pigs immunized with a PCV2b vaccine showed more effective protection against a PCV2a and PCV2b co-challenge than did those immunized with a PCV2a-based vaccine (Opriessnig et al., 2013). In a recent study, we found that a PCV2d-based vaccine significantly reduced PCV2 viremia more than a commercial PCV2a vaccine when applied to pigs naturally infected with PCV2d (Kim and Hahn, 2021). For this reason, the development of a novel PCV2d-based vaccine is required to elicit complete protection against PCV2d infection. Therefore, here, we have evaluated the protective efficacy of a PCV2d-based VLP vaccine in specific pathogen-free (SPF) miniature pigs against an experimental PCV2d challenge.

## 2 Materials and methods

### 2.1 PCV2d-based VLP

The recombinant PCV2d capsid protein (Bac-2dCP) was expressed using the ExpiSf Baculovirus Expression System (Kim and Hahn, 2021). Briefly, *Spodoptera frugiperda* (Sf9) cells (ExpiSf9™, Gibco, United States) were cultured in ExpiSf CD medium (Gibco, United States) and infected with the recombinant baculovirus-expressing capsid protein of PCV2d (GenBank Accession No. KY810325). After 7 days, infected cells were centrifuged at 500 × *g* for 10 min and the pellet dissolved in lysis buffer (50 mM NaH_2_PO_4_ and 300 mM NaCl) containing 1% Igepal CA-630 (Sigma-Aldrich, United States). The supernatant was purified by anion exchange chromatography using Q-Sepharose Fast Flow (GE Healthcare, United States). The purified protein was filtered through a 0.22 μm cellulose acetate membrane (Corning, United States), and protein concentration was measured using a Pierce bicinchoninic acid protein assay kit (Thermo Fisher Scientific, United States). The self-assembled VLPs were observed using a transmission electron microscope (JEM-2100F; JEOL, Tokyo, Japan) at the Chuncheon Center of the Korea Basic Science Institute.

### 2.2 Immunization and the PCV2d challenge

**Figure 1:**
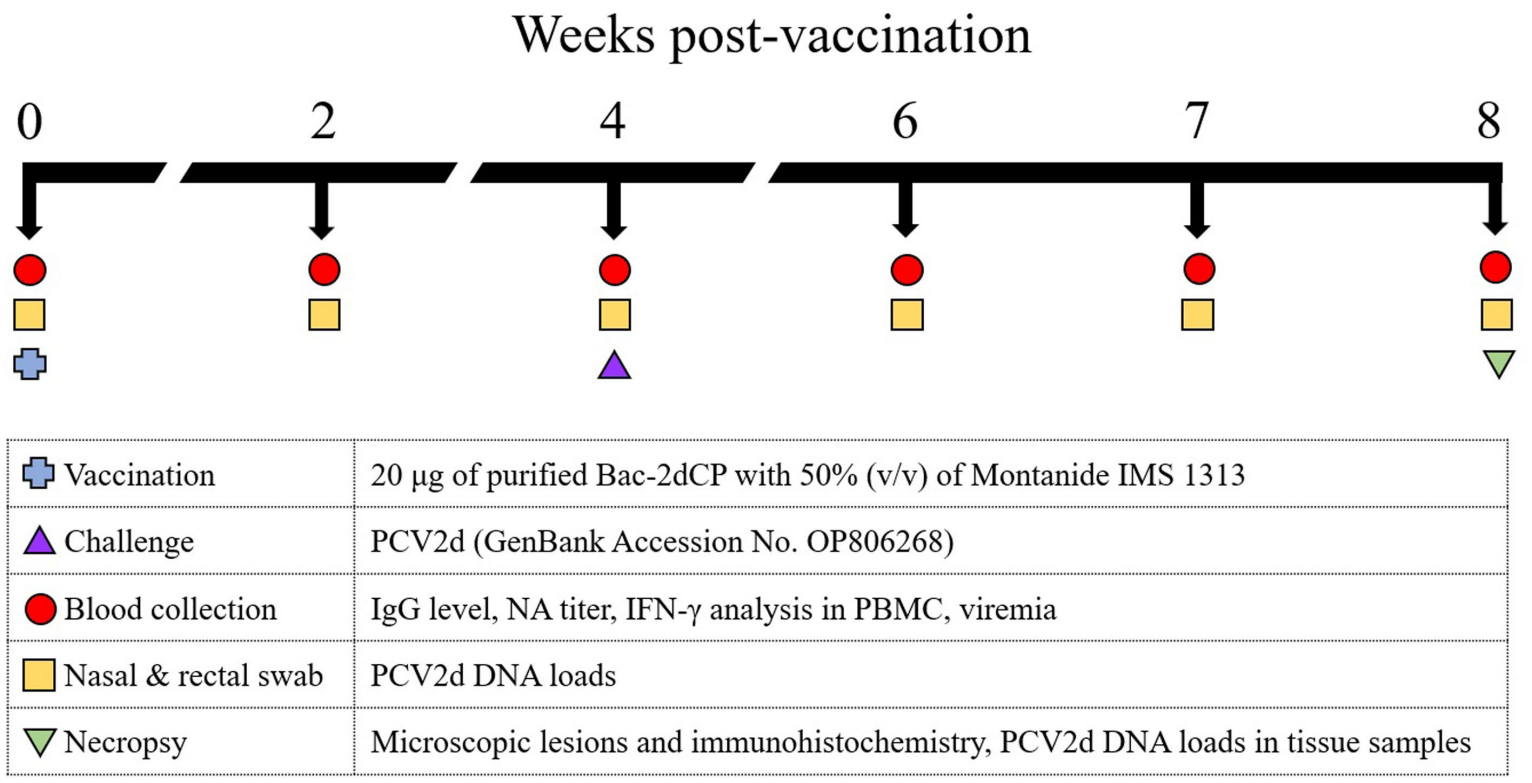
Experimental design. Ten 3-week-old SPF miniature pigs were randomly divided into three groups: The Bac-2dCP (*n* = 5), PC (*n* = 3) and NC (*n* = 2). The Bac-2dCP group was vaccinated intramuscularly once in the neck region. After 4 weeks, Bac-2dCP and PC groups were challenged intranasally with 1 mL of PCV2d (10^5.5^ TCID_50_/mL) in each nostril. The NC group was unchallenged.

The procedures for animal handling, care, and experimental protocols were approved by the Institutional Animal Care and Use Committee of Kangwon National University (Permit No. KW-210510-1). The animal experiment was carried out by Optipharm Medipig (Osong, Korea) in its Biosecurity Level 3 facility. Ten 3-week-old SPF Yucatan miniature pigs were randomly divided into three groups. The Bac-2dCP (*n* = 5) group received a 1 mL dose containing 20 μg of purified Bac-2dCP with 50% (v/v) of Montanide IMS 1313 (Seppic, France) by intramuscular administration in the neck region. At 4 weeks post-vaccination (WPV), the Bac-2dCP and positive control (PC; *n* = 3) groups were challenged intranasally with 1 mL of PCV2d (10^5.5^ 50% tissue culture infective dose, TCID_50_/mL) (GenBank Accession No. OP806268) in each nostril. A third group, the negative control (NC; *n* = 2), was unchallenged. Serum, nasal and rectal swab samples were collected at 0, 2, 4, 6, 7, and 8 WPV (Figure 1). At 4 weeks after the challenge, all animals were humanely euthanized by intravenous injection of 2 mmol/kg potassium chloride solution and necropsied to evaluate pathological lesions and viral DNA loads in lung, lymph node, and kidney.

### 2.3 Clinical signs and average daily weight gain (ADWG)

Following vaccination at 0–7 days, all pigs were monitored daily for rectal temperature and clinical symptoms using a scale ranging from 0 (normal) to 3 (severe). Body weights were measured weekly during the experimental period. ADWG (g/day) was calculated before (0–4 WPV) and after (4–8 WPV) the PCV2 challenge.

### 2.4 Quantification of PCV2 DNA

Viral DNA from the serum, swab, and tissue samples was extracted using a Viral DNA/RNA Extraction Kit (iNtRON Biotechnology, Korea) according to the manufacturer’s protocol. Quantitative polymerase chain reaction (qPCR) assays were conducted using a TOPreal™ qPCR 2X PreMIX (SYBR Green High ROX; Enzynomics, Korea) as described previously (Kim and Hahn, 2021).

### 2.5 Serological assay

All serum samples were tested for PCV2b- and PCV2d-specific IgG antibodies using an indirect enzyme-linked immunosorbent assay (ELISA) (Kim and Hahn, 2021). Microplates (96-well Nunc Maxisorp, Roskilde, Denmark) were coated with 100 ng/well of purified Bac-2bCP or Bac-2dCP. Absorbances were read at 450 nm using a microplate reader (BioTek, United States).

Viral neutralization (VN) titers were determined using an indirect immunofluorescence assay test (Kim et al., 2020). PCV2b supplied by ChoongAng Vaccine Laboratories (Daejeon, Korea) and PCV2d (GenBank Accession No. OP806268) were used in this assay. Briefly, all serum samples from each group were inactivated by heating at 56°C for 30 min. The inactivated samples were serially diluted twofold from 1:4 to 1:16,384 and added to 200 TCID_50_ of PCV2b and PCV2d virus. The serum–virus mixture was incubated at 37°C for 1 h with 5% CO_2_ and then applied to a 70–80% confluent monolayer of PK-15 cells at 37°C for 72 h with 5% CO_2_. VN titers were determined as the highest serum dilution that exhibited >90% neutralization.

### 2.6 IFN**-**γ analysis

Peripheral blood mononuclear cells (PBMCs) were isolated by Ficoll–Hypaque (Choi et al., 2019) density gradient centrifugation at 4, 6, and 8 WPV. PBMCs were stimulated with 10 μg/mL of purified Bac-2dCP for 72 h and the culture supernatants harvested. To determine the PCV2-specific gamma interferon (IFN**-**γ) level, a Porcine IFN-γ ELISA kit (Invitrogen, United States) was used according to the manufacturer’s protocol.

### 2.7 Microscopic lesions and immunohistochemistry

After euthanasia at 8 WPV, lung, lymph node, and kidney tissues were fixed in 10% buffered formalin, embedded in paraffin, and cut into 4 μm sections. After staining with hematoxylin and eosin, microscopic images were obtained through an Olympus BX53 microscope (Olympus, Japan) and analyzed with Olympus cellSens software. Lesions were scored blind as 0 (no lesions), 1 (minimal), 2 (mild), 3 (moderate), or 4 (severe) (Opriessnig et al., 2004).

PCV2 antigen determination in paraffin-embedded sections was performed by immunohistochemical (IHC) analysis using a rabbit PCV2 capsid polyclonal antibody (Invitrogen) and a VECTASTAIN Elite ABC Universal Kit (Vector Laboratories, United States) (Chianini et al., 2003).

### 2.8 Statistical analysis

All data are presented as mean ± standard error of the mean (SEM). Statistical data were generated using GraphPad Prism 8.0.1 software (GraphPad Software, La Jolla, CA, United States), and significant differences were determined using one-way analysis of variance followed by Tukey’s multiple comparisons test. *p* values <0.05 were considered statistically significant.

## 3 Results

### 3.1 Clinical symptoms and ADWG

After vaccination, all pigs in the Bac-2dCP group maintained normal body temperature like the NC group, and no clinical symptoms of abscesses, inflammation, epilepsy, anorexia, depression, shock, vomiting, or diarrhea were seen. ADWG showed no significant difference between groups before the challenge (0–4 WPV) (Table 1). After the challenge (4–8 WPV), the highest ADWG was observed in the Bac-2dCP group, while the growth rate was retarded in the PC group.

**Table 1:** Body weight and average daily weight gain.

	Weeks post-vaccination	Group		
		Bac-2dCP	PC	NC
Body weight (kg)	0	3.06 ± 0.43	3.33 ± 0.48	3.05 ± 0.05
	4	5.76 ± 0.53	5.87 ± 0.47	6.10 ± 0.10
	8	9.88 ± 0.94	9.05 ± 0.93	9.45 ± 0.05
ADWG (g)	0–4	96.4 ± 15.5	90.5 ± 11.3	108.9 ± 12.5
	4–8	147.1 ± 20.4	113.7 ± 61.0	119.6 ± 8.9
	0–8	121.8 ± 18.0	102.1 ± 36.2	114.3 ± 10.7

Data are presented as group mean values (± standard error of the mean).

### 3.2 PCV2-specific humoral immune responses

**Figure 2:**
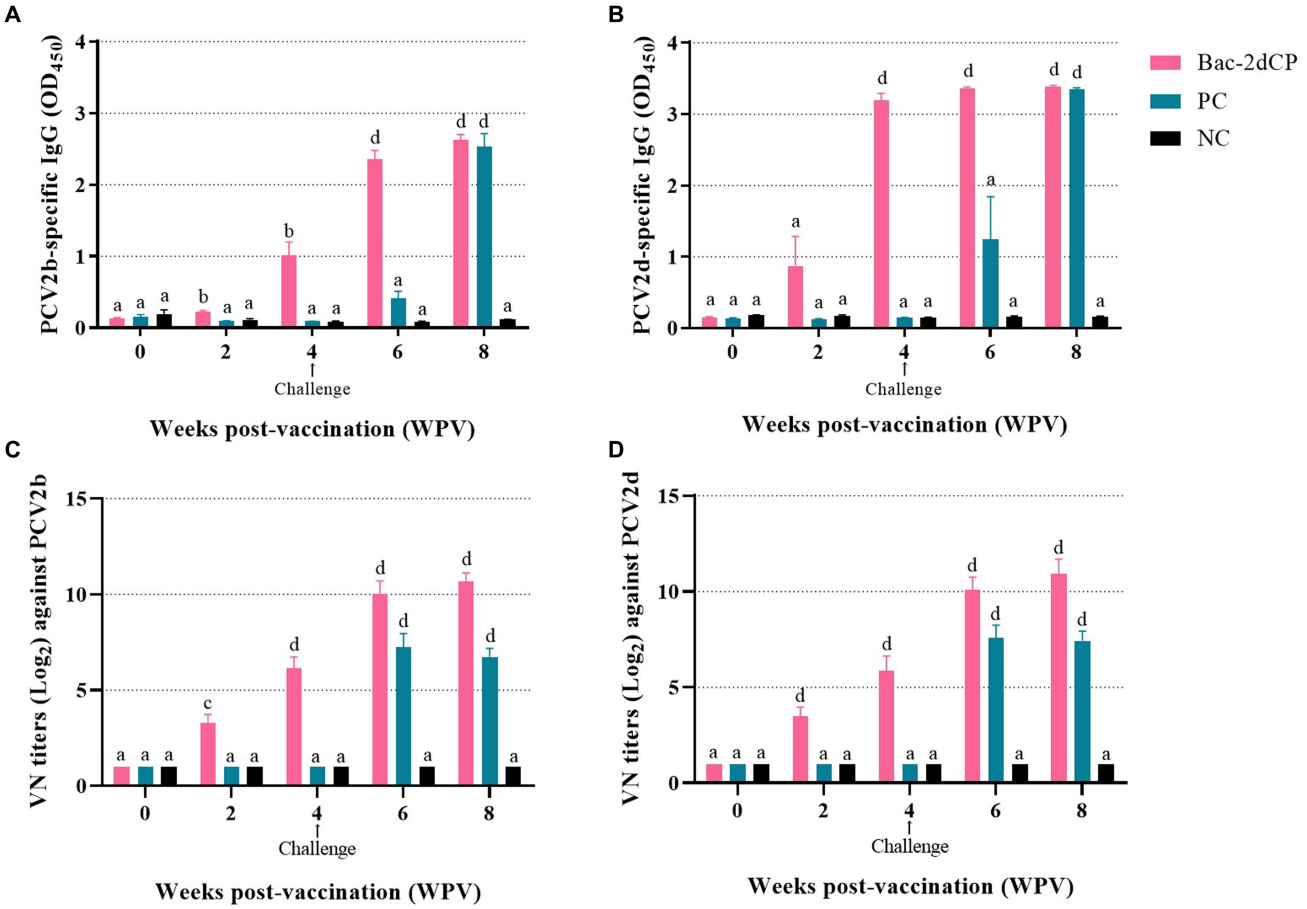
Mean values of serum IgG antibody levels and viral neuralization (VN) titers at different weeks post-vaccination **(A)** PCV2b- and **(B)** PCV2d-specific IgG levels were analyzed by indirect ELISA. VN titers (Log_2_) against **(C)** PCV2b and **(D)** PCV2d were analyzed by indirect immunofluorescence assay (IFA) test. Data were expressed as mean ± standard error of the mean (SEM). Significant differences compared with NC group are indicated by different superscripts (^a^Not significant, ^b^*p* < 0.05, ^c^*p* < 0.01 and ^d^*p* < 0.001).

PCV2-specific maternally-derived antibody was undetected in all SPF miniature pigs at 0 WPV (Figures 2A,B). After vaccination, the Bac-2dCP group seroconverted to PCV2b- and PCV2d-specific IgG antibodies at 2 WPV. Notably, the PCV2-specific IgG levels rapidly increased in the Bac-2dCP group after the challenge and showed significantly higher (*p* < 0.001) values compared with the PC and NC groups at 6 WPV. In addition, the PC group exhibited PCV2-specific IgG levels similar to those of the Bac-2dCP group at 8 WPV, whereas the NC group remained seronegative throughout the experimental period.

### 3.3 Neutralizing activity

Before vaccination, the serum samples in all groups were negative for VN titers against PCV2. In the Bac-2dCP group, PCV2-specific VN titers were first detected at 2 WPV, then greatly increased at 4 and 6 WPV (Figures 2C,D). At 8 WPV, the PCV2b- and PCV2d-specific VN titers reached maximum mean levels of 10.6 and 10.9 log_2_, respectively, which were markedly and significantly higher than those of the NC group (*p* < 0.001). The PC group titers were significantly lower than those of the Bac-2dCP group: *p* < 0.001 for PCV2b and *p* < 0.01 for PCV2d. Nonetheless, their VN titers were substantially higher than those of the NC group.

### 3.4 PCV2 DNA loads

**Figure 3:**
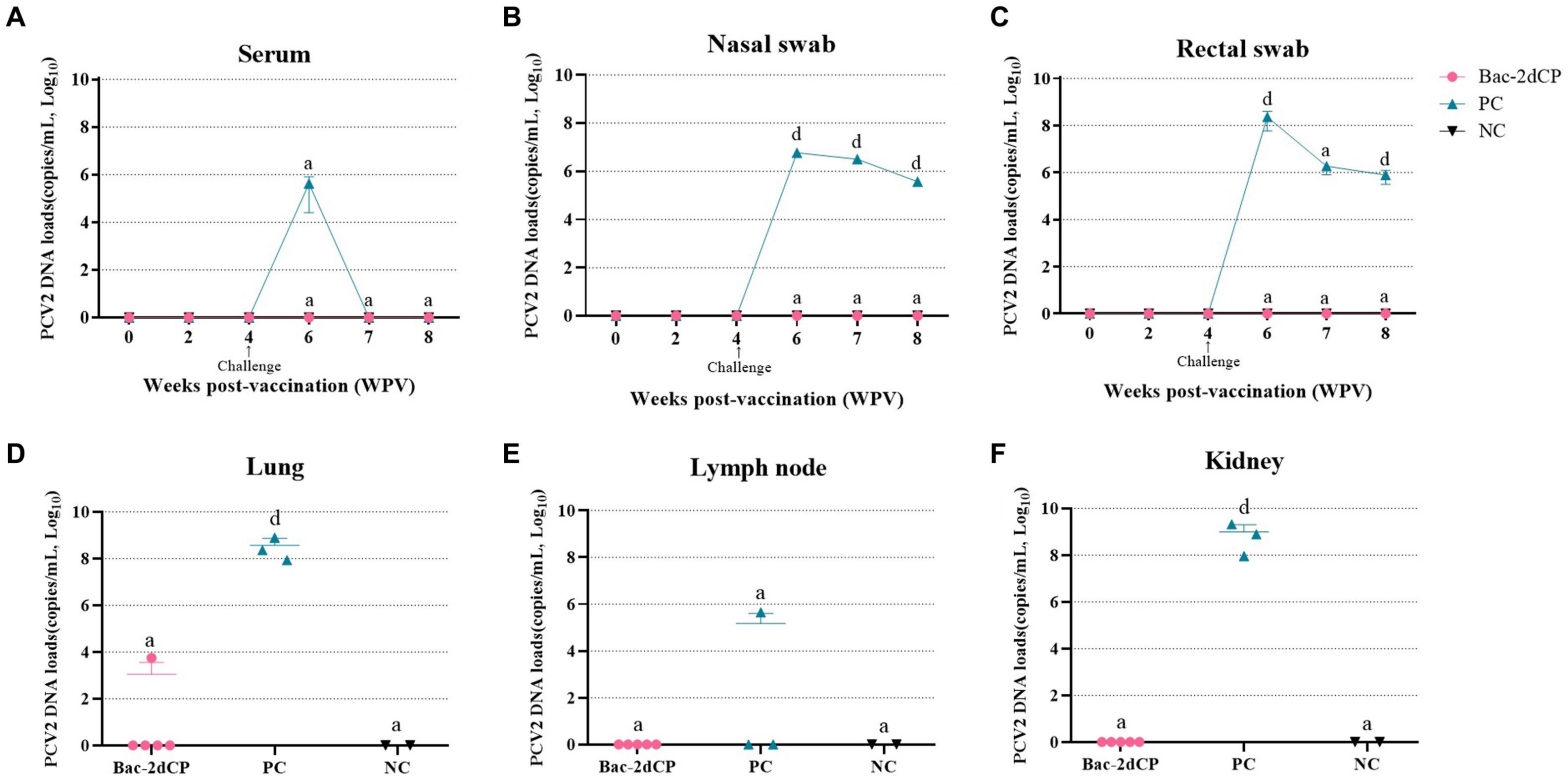
Mean values of the PCV2 genomic copy number in **(A)** serum, **(B)** nasal swab, **(C)** rectal swab, **(D)** lung, **(E)** lymph node, and **(F)** kidney tissue samples at different weeks post-vaccination. PCV2 DNA loads were analyzed by quantitative polymerase chain reaction (qPCR) assays. Data were expressed as mean ± standard error of the mean (SEM). Significant differences compared with NC group are indicated by different superscripts (^a^Not significant and ^d^*p* < 0.001).

In the period from vaccination to challenge, no PCV2d DNA copies were detected in serum, nasal swab, or rectal swab samples from all groups. After 2 weeks post-challenge (6 WPV), a significantly increased level of PCV2d DNA copies in the PC group was observed in the following samples: serum (5.6 log_10_ copies/mL), nasal swab (6.9 log_10_ copies/mL, *p* < 0.001), and rectal swab (8.3 log_10_ copies/mL, *p* < 0.001) (Figures 3A–C, respectively). Importantly, the PC group showed a significant amount of PCV2d DNA in lung (8.5 log_10_ copies/mL, *p* < 0.001), lymph node (5.1 log_10_ copies/mL), and kidney tissue samples (8.9 log_10_ copies/mL, *p* < 0.001) (Figures 3D–F, respectively). By contrast, PCV2d DNA was not detected in any serum, swab, or tissue (except one lung) samples from the Bac-2dCP group, and in this respect, the Bac-2dCP group was closely similar to the NC group.

### 3.5 IFN**-**γ levels

At 4 WPV, production of IFN-γ in PBMC stimulated with Bac-2dCP was undetected in the PC and NC groups (Table 2). However, the Bac-2dCP group showed a significantly higher (*p* < 0.05) secretion level of IFN-γ compared with the PC and NC groups. By 2 weeks post-challenge (6 WPV), the PCV2d-specific IFN-γ level of the Bac-2dCP group had decreased, but was still markedly higher than those of the PC and NC groups at 8 WPV.

**Table 2:** Mean group interferon-gamma (IFN-γ) in PBMC.

Group	Weeks post-vaccination		
	4	6	8
Bac-2dCP	128.1 ± 54.7^b^	11.4 ± 6.6^a^	72.9 ± 31.3^a^
PC	0.0^a^	12.8 ± 5.9^a^	22.8 ± 22.3^a^
NC	0.0^a^	3.6 ± 3.6^a^	7.3 ± 7.3^a^

The data are presented as group mean values (pg/mL ± standard error). Significant differences (*p* < 0.05) compared with NC group are indicated by different superscripts.

### 3.6 Histopathological and immunohistochemical results

**Figure 4:**
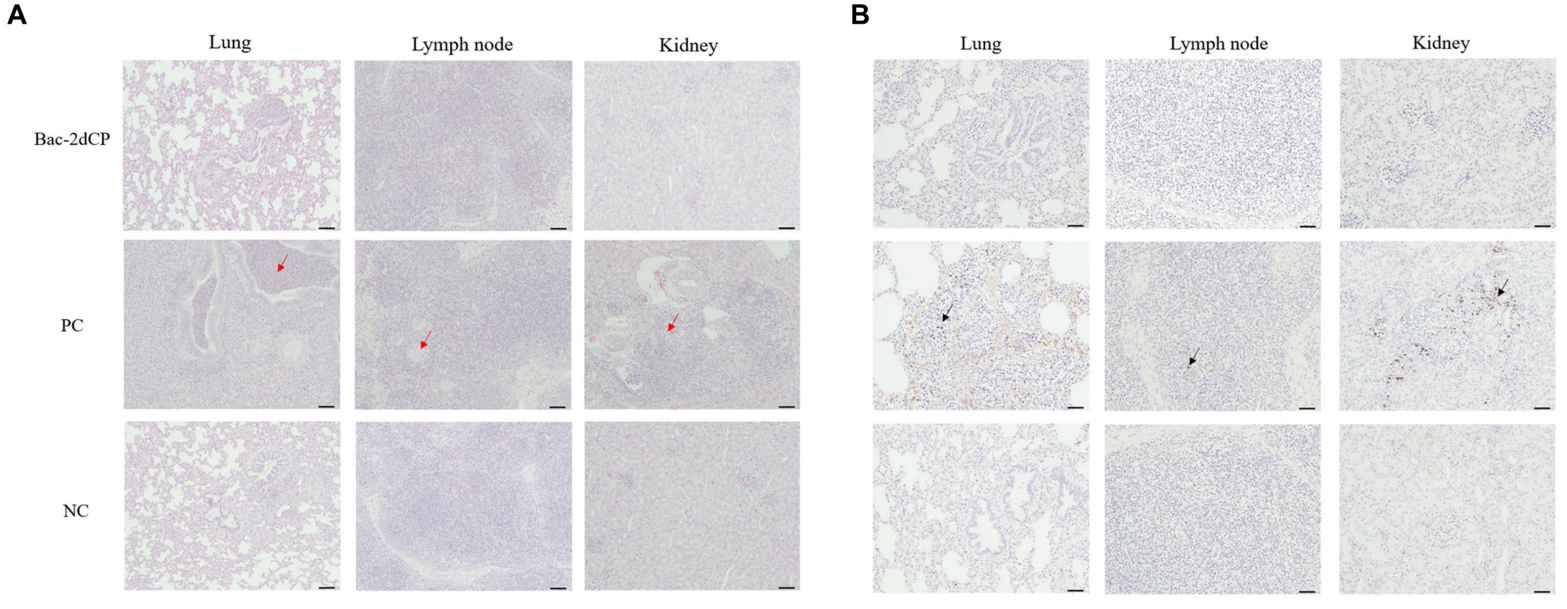
Histopathological evaluation and detection of PCV2 antigen in tissues at 4 weeks post-challenge. **(A)** Hematoxylin and eosin staining and **(B)** immunohistochemistry in lung, lymph node, and kidney tissues. The red arrows indicate pulmonary edema, lymphoid depletion and interstitial inflammatory cell infiltration in the lung, lymph node and kidney tissues of PC group, respectively. PCV2 antigens were shown by black arrows. Scale bars indicate **(A)** 100 μm and **(B)** 50 μm.

**Figure 5:**
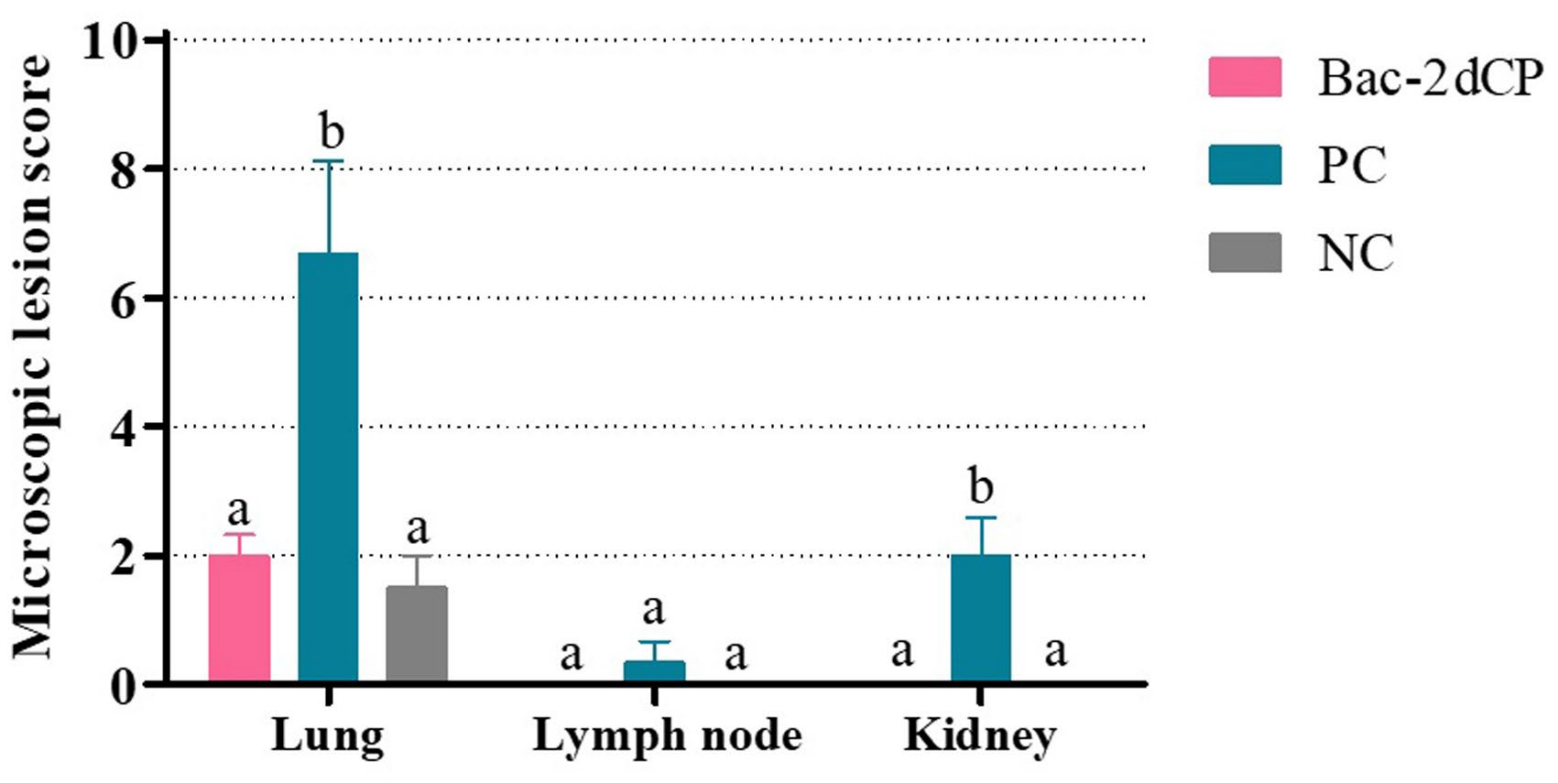
Microscopic lesion scores in lung, lymph node, and kidney at 4 weeks post-challenge. Histopathological lesions were scored blind as 0 (no lesions), 1 (minimal), 2 (mild), 3 (moderate), or 4 (severe). Data were expressed as mean ± standard error of the mean (SEM). Significant differences compared with NC group are indicated by different superscripts (^a^Not significant and ^b^*p* < 0.05).

Mild perivascular and peribronchiolar cuffing were observed in lesions that were found in all groups (Table 3 and Figure 4A). Some pigs in the Bac-2dCP and PC groups showed bronchus-associated lymphoid tissue (BALT) hyperplasia. Notably, the PC group showed PCV2-associated lung lesions of suppurative bronchointerstitial pneumonia, peribronchiolar fibroplasia with bronchiolar segmentation, and pulmonary edema. In addition, lymphoid depletion in lymph node and interstitial inflammatory cell infiltration in kidney were observed only in the PC group. However, the Bac-2dCP group did not display PCV2-associated lesions in lung, lymph node, or kidney, a result identical to that of the NC group. Interestingly, lung and kidney lesion scores in the Bac-2dCP group were significantly (*p* < 0.01) lower than those in the PC group (Figure 5).

**Table 3:** Histopathologic and IHC findings in lung, lymph node, and kidney.

Findings	No. of pigs (positive/total)		
	Bac-2dCP	PC	NC
*Lung*			
Perivascular and peribronchiolar cuffing	5/5	3/3	2/2
BALT hyperplasia	2/5	1/3	0/2
Suppurative bronchointerstitial pneumonia	0/5	1/3	0/2
Peribronchiolar fibroplasia with bronchiolar segmentation	0/5	1/3	0/2
Pulmonary edema	0/5	2/3	0/2
PCV2 detection (IHC)	1/5	1/3	0/2
*Lymph node*			
Lymphoid depletion	0/5	1/3	0/2
PCV2 detection (IHC)	0/5	2/3	0/2
*Kidney*			
Interstitial inflammatory cell infiltration	0/5	3/3	0/2
PCV2 detection (IHC)	0/5	3/3	0/2

For the IHC examination, PCV2 antigen was undetected in almost all lung, lymph node, and kidney tissue samples in the Bac-2dCP and NC groups (Figure 4B). However, the PC group showed high amounts of PCV2 antigen in tissue samples.

## 4 Discussion

Following the introduction of commercial PCV2a-based vaccines, new genotypes have emerged by viral evolution (Ilha et al., 2020). To alleviate these concerns, cross-protective ability is an important quality of PCV2 vaccines. Our previous study demonstrated that our Bac-2dCP VLP vaccine provides not only effective protection against the homologous PCV2d genotype (the most dominant genotype), but also cross-immunization protection against the heterologous PCV2b genotype in pigs naturally infected with PCV2d (Kim and Hahn, 2021). Arising from this result, the objective of the present study was to evaluate the protective efficacy of Bac-2dCP VLP vaccine in an SPF miniature pig model against an experimental PCV2d challenge. The SPF miniature pig provides the advantages of no maternal antibodies, genetic stability, susceptibility to infection, ease of rearing, and higher statistical power in vaccination and challenge experiments (Khan, 1984; Klinkenberg et al., 2002; Gan et al., 2020).

The presence of neutralizing antibody against PCV2 is an important mechanism to control PCVAD and has a pivotal role in viral clearance (Chae, 2012; Dvorak et al., 2018). A previous study reported that a PCV2a-based commercial vaccine induced neutralizing antibody titers of 8.0 log_2_ at 7 WPV in conventional pigs, and that PCV2 DNA was detected at low levels in blood after PCV2a, PCV2b, and PCV2d challenges (Park et al., 2019). In addition, commercial PCV2a-vaccinated herds showed a reduction of PCV2 viremia, shedding, and transmission against a PCV2d challenge under experimental conditions (Opriessnig et al., 2017). However, low levels of PCV2 viremia mean that the virus has not completely cleared, resulting in chronic subclinical infection with PCV2. Therefore, these commercial PCV2 vaccines appear to provide incomplete cross-protection against the current dominant PCV2d genotype (Peswani et al., 2022). Consequently, it is important that next-generation PCV2 vaccines induce sufficient immune protection against PCV2d. A recent study demonstrated that vaccination with PCV2d VLP in pigs induced high levels of PCV2d-specific neutralizing antibodies, and that PCV2 DNA loads in blood and nasal swab against PCV2d and PRRSV dual-challenge were similar to those of the unchallenged group (Kang et al., 2021). In the present study, miniature pigs vaccinated with Bac-2dCP VLP elicited a sufficient immune response in PCV2b and PCV2d VN titers. Interestingly, the vaccinated group was confirmed to be almost devoid of PCV2, unlike the significantly higher viral loads from serum, swab, and tissue samples in the PC group after the PCV2d challenge. Further, the vaccinated group exhibited almost complete protection against PCV2-associated microscopic lesions in lung, lymph node, and kidney, similar to the results in the NC group. These data suggest that sufficient neutralizing antibodies produced by vaccination contributed to effective protection against PCV2d infection, reduced PCV2-associated pathological lesions. Meanwhile, histopathologic difference against PCV2d challenge in PC group seems to be dependent on the susceptibility or innate immunity between the individual pig.

Cell-mediated immunity is also known to be a key factor in PCV2 clearance and long-term protection against PCV2 infection (Venegas-Vargas et al., 2021). PCV2-specific IFN-γ production is related to reductions in viremia, shedding, and PCV2-associated lesions (Ferrari et al., 2014; Park et al., 2014). A previous study reported that the maximum PCV2-specific IFN-γ level in PBMC was 74.4 pg/mL in pigs vaccinated with Ingelvac CircoFLEX at 3 WPV (Li et al., 2020). In the present study, a high level of IFN-γ response was observed in the vaccinated group (128.1 pg/mL) at 4 WPV, and was significantly different (*p* < 0.05) from the two control groups. After challenge, the IFN-γ of vaccinated group was again elevated at 8 WPV, it could be inferred that PCV2-specific IFN-γ was secreted by memory T cells to protect against PCV2d shedding from nasal and rectal of PC group. This enhanced IFN-γ secretion by vaccination appears to regulate the protective immune response and to contribute to viral clearance in serum, swab, and tissue samples after a PCV2d challenge.

## 5 Conclusion

The present study demonstrated that recombinant Bac-2dCP VLP vaccine can effectively induce PCV2-specific humoral and cell-mediated immune responses and provide complete protection from PCV2 viremia, nasal, and rectal shedding. It can also significantly reduce viral loads in lung, lymph node, and kidney tissues against a PCV2d challenge in SPF miniature pigs. Therefore, the Bac-2dCP vaccine is an attractive candidate to control the PCVAD caused by the currently prevalent PCV2d genotype. However, further studies are needed to evaluate the comparative efficacy and immune persistence of Bac-2dCP vaccine in conventional pigs.

## Data availability statement

The original contributions presented in the study are included in the article/Supplementary material, further inquiries can be directed to the corresponding author.

## Ethics statement

The animal studies were approved by Institutional Animal Care and Use Committee of Kangwon National University. The studies were conducted in accordance with the local legislation and institutional requirements. Written informed consent was obtained from the owners for the participation of their animals in this study.

## Author contributions

KK: Writing – original draft, Writing – review & editing. KC: Writing – review & editing. MS: Writing – review & editing. T-WH: Writing – review & editing.
